# Exploring Associations between Susceptibility to the Use of Electronic Nicotine Delivery Systems and E-Cigarette Use among School-Going Adolescents in Rural Appalachia

**DOI:** 10.3390/ijerph17145133

**Published:** 2020-07-16

**Authors:** Hadii M. Mamudu, Christen Nwabueze, Florence M. Weierbach, Joshua Yang, Antwan Jones, Michelle McNabb, Esther Adeniran, Ying Liu, Liang Wang, Cynthia J. Blair, Adeola Awujoola, David L. Wood

**Affiliations:** 1 Department of Health Services Management and Policy, College of Public Health, East Tennessee State University, Johnson City, TN 37614, USA; ZCJB7@etsu.edu; 2 Department of Biostatistics and Epidemiology, College of Public Health, East Tennessee State University, Johnson City, TN 37614, USA; NWABUEZE@etsu.edu (C.N.); ADENIRANE@etsu.edu (E.A.); liuy09@etsu.edu (Y.L.); wangl2@etsu.edu (L.W.); AWUJOOLA@etsu.edu (A.A.); 3 Department of Graduate Studies, College of Nursing, East Tennessee State University, Johnson City, TN 37614, USA; WEIERBACH@etsu.edu; 4 Department of Public Health, California State University, Fullerton, CA 92831, USA; jsyang@fullerton.edu; 5 Department of Epidemiology, Milken School of Public Health, and Department of Sociology, The George Washington University, Washington, DC 20052, USA; antwan@gwu.edu; 6 Northeast Regional Office, Tennessee Department of Health, Johnson City, TN 37604, USA; Michelle.McNabb@tn.gov; 7 James H. Quillen College of Medicine, East Tennessee State University, Johnson City, TN 37604, USA; WOODDL@etsu.edu

**Keywords:** electronic nicotine delivery systems, e-cigarettes, Appalachia, adolescents, rural susceptibility, social determinants of health

## Abstract

Electronic nicotine delivery systems (ENDS) use, including e-cigarettes, has surpassed the use of conventional tobacco products. Emerging research suggests that susceptibility to e-cigarette use is associated with actual use among adolescents. However, few studies exist involving adolescents in high-risk, rural, socioeconomically distressed environments. This study examines susceptibility to and subsequent usage in school-going adolescents in a rural distressed county in Appalachian Tennessee using data from an online survey (*N* = 399). Relying on bivariate analyses and logistic regression, this study finds that while 30.6% of adolescents are ever e-cigarette users, 15.5% are current users. Approximately one in three adolescents are susceptible to e-cigarettes use, and susceptibility is associated with lower odds of being a current e-cigarette user (OR = 0.03; CI: 0.01–0.12; *p* < 0.00). The age of tobacco use initiation was significantly associated with decreased current use of e-cigarettes (OR = 0.89; CI: 0.83–0.0.97; *p* < 0.01). Overall, the results of this exploratory study suggest the need for larger studies to identify unique and generalizable factors that predispose adolescents in this high-risk rural, socioeconomically disadvantaged region to ENDS use. Nevertheless, this study offers insight into e-cigarette usage among U.S adolescents in rural, socioeconomically disadvantaged environments and provides a foundation for a closer examination of this vulnerable population.

## 1. Introduction

The use of electronic nicotine delivery systems (ENDS) [1] has proliferated in the United States (USA) across demographic, socioeconomic, and geographic characteristics. While there has been an uptake of ENDS across all age groups, recent reports from the National Youth Tobacco Survey (NYTS) [2,3,4] show that the use of ENDS among middle and high school students has surpassed the use of conventional tobacco products (CTPs) such as cigarettes. According to one study, the overall ENDS use among USA middle and high school students was 20.0% (10.5% middle school, 27.5% high school in 2019) [2]. This increase in ENDS use threatens the national gains in tobacco control over the past 50 years [5] because: (1) emerging studies have found an uptake of ENDS use among never-smoking youth [2,3], (2) ENDS use is associated with subsequent use of CTPs [6,7,8,9], and (3) it is associated with dual- and poly-use in youth [10,11,12]. ENDS use also renormalizes smoking and undermines the denormalization efforts of tobacco control, especially amidst weak regulatory regimes for ENDS, compared to CTPs [5,13,14,15,16]. Over 90% of regular users of all tobacco products, including ENDS, started as youth [17,18,19] and are targeted by the tobacco industry through advertising, marketing, and promotions [19]. Research shows that adolescence is a period of vulnerabilities and risk taking as perceptions of risks do not stop adolescents from indulging in risky behaviors, and that adolescents are predisposed to risk taking behaviors due to factors such as greater experience of emotional satisfaction with risk-taking behavior, exploratory drive, and less adult supervision [20,21,22]. In this regard, addressing the use of ENDS among adolescents is critical to preventing them from initiating tobacco use, which continue to be the leading preventable cause of morbidity and mortality in the U.S [5,23,24]. Moreover, it has been established that nicotine, including those from ENDS, has adverse effects on the brain development of youth [5,19,24], and increasing evidence indicates that ENDS use may be associated with acute lung injuries [25]. As such, identifying and targeting at-risk youth to inform the development of interventions is significant to efforts to prevent tobacco use initiation among youth and the morbidity and mortality risks associated with ENDS use [26].

Research suggests that tobacco use is a learned behavior [27] and that susceptibility to smoking, defined as a lack of a firm commitment to abstain from smoking, is a risk factor for actual smoking [5,19,24,28,29,30]. Since Pierce et al. [31] developed an index to ascertain smoking susceptibility in the 1990s, several studies in the USA, Canada, and Poland [32,33,34,35] have attempted to understand the association between susceptibility and tobacco use initiation in youth [36,37,38,39,40], young adults [41,42], and adults [43]. Research studies have found that this susceptibility index is valid and reliable for ascertaining susceptibility to ENDS use. Recent studies using this ENDS smoking susceptibility index found that it is associated with initiation of actual use [2,3,36,37]. Wang et al. found that 45% of never users of tobacco products were susceptible to e-cigarette use and increased ENDS use [2]. Using cross-sectional data from the Wave 1 of the Population Assessment and Tobacco and Health (PATH) Study (2013–2014), Trinidad et al. found similar evidence, with 27.4% of participants aged 12–17 years identified to be susceptible to e-cigarettes use [36]. In a longitudinal study of middle and high school students over 6 months, e-cigarette use susceptibility was associated with a significant increase in the likelihood of initiation of e-cigarette use by over four times and past 30-day e-cigarette use by over five times [44]. These studies have treated susceptibility as either an outcome variable [37,45,46,47,48] or exposure variable [36,38]. The studies involving susceptibility as the outcome variable reveal that marketing, advertising, and promotion increase susceptibility for both the use of CTPs [39,45,46,47,49,50,51,52] and ENDS use [2,36,44,49]. Much of the susceptibility literature in the USA is centered on CTPs [37,39,45,47], although studies pertaining to ENDS use [2,36,37] continue to emerge within population subgroups [44,49]. The impetus for this study is the paucity of studies involving youth in rural areas, such as those in the high-risk environment of Central Appalachia [53,54,55,56,57,58].

This study aimed to explore the associations between susceptibility to e-cigarette use and ever and current use of e-cigarettes among youth in a rural county in Appalachian Tennessee, which was designated as distressed by the Appalachian Regional Commission (ARC). The Central Appalachian region is comprised of 230 non-contiguous counties in Kentucky, North Carolina, Ohio, Tennessee, Virginia, and West Virginia. The region is predominantly rural and mountainous, with a history of both tobacco use and being culturally resistant to outside influences [59,60]. The counties have a disproportionately high prevalence of tobacco use, tobacco-induced diseases, and limited programs for tobacco control, which makes them part of the “Tobacco Nation” [53]. Additionally, ARC has designated over 90% of the counties in the region as at-risk or economically distressed, thereby exemplifying how social determinants of health (SDOH) may place underserved populations at risk for adverse health conditions [61,62,63,64,65]. This combination of contextual factors has created an environment that predisposes youth to the use of tobacco products, including ENDS. Thus, coupling the disparities in tobacco use [4,19,66,67] with increasing evidence that ENDS have “gateway” effects because non-smokers that initiate use transition to use CTPs [68,69,70,71,72,73,74] and other substances [75] provided the rationale for focusing on adolescents in Central Appalachia. This study is well-positioned to inform targeted intervention efforts by identifying factors that lead to ENDS use, which could prevent ENDS initiation and help to address the burden of tobacco use in the region. 

## 2. Methods

### 2.1. Study Population 

The study population involves youth in 8th and 9th grades in a school located in an ARC-designated distressed rural county in Appalachian Tennessee [76]. In collaboration with the school, the County Health Department and the Regional Health Department conducted an online survey administered in March/April 2019. The survey collected data on the prevalence, knowledge, opinions, and perceptions about tobacco product use, including ENDS. While passive consent procedure was used to obtain parental/guardian/caregiver consent, in compliance with the Helsinki Declaration [77], the youth participants provided assent, voluntarily participated in the study, and were informed that they could withdraw anytime during the study without repercussions. Additionally, as part of the agreement between the health department, the school administration, and the parent’s association, participation in the study was anonymous with the survey questionnaire having limited demographic questions. This strong collaboration between the health department, school administration, parents’ association, and the researchers culminated in 92% response rate. The data used in this study was the de-identified survey file, which was sent to the researchers/investigators. 

The Institutional Review Board of East Tennessee State University (ETSU) has approved the analysis of this de-identified data from the health department for publication.

### 2.2. Measurements

Extensive discussions between the Regional Health Department, the County Health Department, the school administration, and the parents’ association culminated in a 30-item questionnaire generated by the research team using standard and validated questions from the National Youth Tobacco Survey (NYTS) [24] and Global Youth Tobacco Survey (GYTS) [25,26]. This questionnaire comprised of questions on the use of CTPs (e.g., cigarettes) and ENDS (e.g., e-cigarettes), access to CTP/ENDS, susceptibility to CTP and ENDS use, attitudes/beliefs about CTP/ENDS use, exposure to the use of CTPs and ENDS (including secondhand smoke/aerosol), access to industry merchandise, perceptions about dangers of CTP/ENDS use, peer and familial CTP/ENDS use behavior, exposure to CTP/ENDS advertising, exposure to CTP/ENDS via social media, and demographic information. All responses to the questions in the questionnaire were self-reported. 

#### 2.2.1. Outcome Variable: Ever Use of ENDS and Current Use of ENDS

ENDS in this study includes e-cigarettes and JUUL, as described by the USA Food and Drug Administration [1]. JUUL, the most common e-cigarette brand, accounting for over 50% of the market share as of 2019 [78], was included in the question because emerging reports suggest that youth do not usually consider JUUL (“Juuling”) as an e-cigarette (commonly associated with vaping) [79,80]. Thus, the survey questionnaire captured the totality of ENDS used by adolescents in the U.S. The ever use of ENDS was measured with the question “Have you ever tried electronic cigarettes, including JUUL, even just one or two puffs?” (Yes/No), while the current use of ENDS was ascertained with two questions: (1) During the past 30 days, how many days did you use the following tobacco products? (list of products adapted from the NYTS [2,4], the USA Centers for Disease Control and Prevention (CDC) and the FDA [1], including JUUL); and (2) During the past 30 days, how many days did you use the products in the previous question: please list the product and number of days. These two questions were combined to identify the current users of ENDS in our study population. Ever use of ENDS and current use of ENDS, were measured as binary variables: “0” for “No” and “1” for “Yes”.

#### 2.2.2. Exposure Variable: Susceptibility to END Use

The focal independent variable for this study was susceptibility to ENDS use, a key determinant of its initiation [2,30,49]. Adapting Pierce et al. [30,31] we utilized two questions to ascertain ENDS susceptibility: (1) If one of your best friends offered you an electronic cigarette or vapor product, would you smoke it? (Definitely yes, probably yes, probably not, definitely not); and (2) Do you think that you will try an electronic vapor product (including JUUL) any time in the next year? (Definitely yes, probably yes, probably not, definitely not). The responses for “Definitely yes” and “Probably yes” were recoded as “yes” (i.e., susceptibility to ENDS use) and “Probability not” and “Definitely not” were recoded as “no” (i.e., not susceptible to ENDS use). Respondents who indicated “yes” to either question were classified as being susceptible to ENDS use, which suggests that those susceptible to ENDS use were non-users when the survey was administered. Therefore, susceptibility to ENDS use was measured as a binary variable: “0” for “not susceptible” and “1” for “susceptible”.

#### 2.2.3. Covariates

The variables to assess ENDS use were based on SDOH, selected from literature [2,19,81], and guided by the socioecological model promoted in the National Cancer Institute’s (NCI) Monograph 22 [82]. These variables include sex, current age of participant, age of tobacco use initiation, school grade, perception of tobacco products as dangerous, and parental/guardian advise against tobacco use (including ENDS). For sex, male was coded as the referent category. The current age of participant was assessed as “How old are you?” with the response as a continuous variable of age in years. The age of tobacco use initiation was ascertained with the question, “How old were you when you first tried a tobacco product?” as a continuous variable. School grade was limited to 8th and 9th grade; however, it was dropped from the regression model because of the correlation with age. Perception of danger of all tobacco products was ascertained with “How strongly do you agree or disagree with the statement: “All tobacco products are dangerous?” with Likert-scale responses varying from strongly disagree to strongly agree. This variable was recoded as “agree” or “disagree”, with the former (i.e., strongly agree and agree) being the referent category. Access to tobacco products was ascertained with “How easy would it be for you to get tobacco products (including ENDS) if you wanted them?” The responses were “very easy”, “somewhat easy”, and “not easy”. These responses were recoded as “1” (Easy, i.e., “very easy” + “somewhat easy”) and “0” (not easy). Parental/guardian advise against tobacco use was ascertained with “During the past 12 months, did your parents or guardians talk to you, even for once, about not using any type of tobacco products (including ENDS)?” and was recoded as “0” for “No” and “1” for “Yes”.

### 2.3. Statistical Analysis 

Descriptive analyses (frequencies, percentages) were used to examine the prevalence of ever use of ENDS and current use of ENDS, respectively. Bivariate relationships between each outcome variable and the exposure variable (i.e., susceptibility to ENDS use) as well as all the covariates were analyzed using Pearson’s chi-square test. We further conducted data diagnostics to identify autocorrelation and multi-collinearity, and the results showed the data was adequate for multivariable analysis. As such, multiple logistic regression analyses were performed to delineate the associations of susceptibility to ENDS with the two outcome variables. i.e., ever use of ENDS and current ENDS use, after adjusting for covariates. *p* < 0.05 was considered statistically significant. The adjusted odds ratios (ORs) and the associated 95% confidence intervals (CIs) are reported. All statistical analyses were preformed using SAS version 9.4 (SAS Institute, Cary, NC, USA).

## 3. Results

### 3.1. Characteristics of Study Population

Study population characteristics are shown in Table 1. A total of 399 students participated in the study, 29.1% of which were identified as susceptible to ENDS use. Of all the participating students, 30.6% reported that they were ever users of e-cigarettes and 15.5% indicated that they were current e-cigarette users. About 52.7% (*n* = 185) were females and the overall mean age of the population was 14.8 ± 1.3 years. The mean age of initiation of use of tobacco products was 13.5 ± 1.8 years. While 75.8% of the students perceived that the use of all tobacco products (including ENDS) was dangerous to health, 61.2% reported that access to e-cigarettes was easy. Fifty-one percent of the students reported that they had received advice from parent/guardian not to use any tobacco product. Overall, 65.2% of the students reported that they had never used any tobacco product.

### 3.2. Adjusted Association between Susceptibility to Use and Ever Use of E-Cigarettes 

Table 2 shows the association between susceptibility to e-cigarette use and ever use of e-cigarette was statistically significant after adjusting for covariates (OR = 0.09, 95% CI = 0.03–0.25, *p* < 0.0001). Age at initiation of tobacco use was also significantly associated with decreased likelihood of ever using e-cigarette, with reduced likelihood as age increases (OR = 0.69, CI = 0.67–1.32, *p* < 0.0001).

### 3.3. Adjusted Association between Susceptibility to Use and Current Use of E-Cigarettes

Table 2 shows the results of the multivariable logistic regression analysis of the association between susceptibility to ENDS use and current use of ENDS. The results show that susceptibility to e-cigarette use was associated with decreased likelihood of being a current user of e-cigarettes (OR = 0.03, CI = 0.01–0.12 *p* < 0.0001), compared with those not susceptible. Also, age at tobacco use initiation was significantly associated with decreased likelihood current user (OR = 0.89, CI = 0.83–0.97, *p* = 0.0051). Current age, sex, perception of the danger of tobacco products, beliefs about the ease of access e-cigarettes, and parental/guardian advise against tobacco were not significantly associated with current use of e-cigarette. 

## 4. Discussion

ENDS use among USA youths has surpassed all CTPs since 2014 [2,19,28]. However, studies involving youth in high-risk environments such as distressed rural areas that predispose youth to tobacco use and make them vulnerable to initiate ENDS use are sparse [10,55,56,57,58,83]. This study explored the use of ENDS among students in an ARC-designated, distressed, rural county in Appalachian Tennessee in 2019. While 30.6% of the adolescents had tried e-cigarettes before, 15.5% were current users of e-cigarettes, which is comparable a study in the region [58] but higher than what was found in an earlier Appalachian youth [56], and in an urban setting of Connecticut ^44^. While these differences in the prevalence of ENDS use among Appalachian youth is consistent with the trend observed in national data [2], the prevalence of current e-cigarettes in our study population is less than that of NYTS (20%) conducted in the same year (2019), but it is considerably higher than the use of CTPs in the NYTS [28]. This finding is concerning since emerging evidence indicates that the use of e-cigarettes may be a transition to the use of CTPs [6,7], with the potential to undermine the progress made so far in tobacco control efforts [5,19,24]. Given the role of tobacco in the causation of preventable morbidity and mortality, any effort to prevent initiation of CTPs, including measures to prevent the initiation and transition from e-cigarettes to CTPs, should be encouraged.

Emerging research continues to examine the reasons that contribute to the rapid uptake of ENDS use in the USA [2,19]. This study explored susceptibility to ENDS use and its association with its use (ever and current) after controlling other study variables among adolescents in a school in Appalachian Tennessee. Approximately one in three students was identified to be susceptible to e-cigarette use. The susceptibility to e-cigarette use was statistically significantly associated with ever use of e-cigarettes; but the findings also demonstrate that susceptibility significantly decreased the likelihood of current use of e-cigarettes, which contradicts several national studies [2,52]. For this reason, we examined the questions for the susceptibility index independently and the results were similar, suggesting that the contradictory finding within our study population was not due to data management and measurement errors. This result may be due to the cross-sectional nature of the study and/or unique characteristics of the study population. Nonetheless, the fact that one in three adolescents in this study was identified as susceptible to ENDS use suggests the need for further studies with much larger samples and qualitative components. Further study is necessary in order to explain why these susceptible adolescents in this distressed rural environment were less likely to be current e-cigarette users. 

A myriad of factors has been identified through quantitative and qualitative studies as determinants of ENDS use among youth [2,19,81]. As such, we further explored sociodemographic factors that were selected based on the extant literature (sex, age, perceptions of harm, access to ENDS, and parental/guardian advice) [2,19,81]. While sex was not statistically significant, the age of initiation of tobacco use was significantly associated with decreased current use of e-cigarettes. This finding indicates that a higher proportion of youths began using e-cigarettes at a much younger age, which suggests that early adolescence may be where intervention efforts should be targeted in the prevention of initiation of e-cigarettes. Thus, the implementation of age restriction policies such as the national Tobacco 21 policy that has set the minimum legal sales age at 21 years [84] could delay and prevent the initiation of ENDS use.

The perception of harm of tobacco products is a major factor in the initiation and continuation of the use of tobacco products, including ENDS [2,19,56]. In a survey of Appalachian middle and high school students conducted between 2014 and 2016, it was found that compared to never users, e-cigarette only users were more likely to disagree that smoking and e-cigarettes cause health problems and that e-cigarettes cause addiction [56]. Among this study’s participants who had ever used e-cigarette and current users, 67.3% and 75.5% respectively, agreed or strongly agreed that tobacco products were dangerous to health. However, the results for our study were mixed; while perception that all tobacco products were dangerous to health was not significantly associated with ever e-cigarette use, it significantly decreased the likelihood of being a current ENDS user. These mixed results may have stemmed from how youths perceive the health danger of the use of CTPs in relation to ENDS. Although ENDS are considered non-conventional tobacco products in the USA [1], studies have consistently found that the perceptions that ENDS are less harmful than CTPs such as cigarettes are associated with ENDS use among youth [2,19]. These studies have found that ENDS have been marketed as a ‘healthier alternative’ to tobacco and/or as a way to transition off cigarettes for established smokers. As a consequence, while studies have found that youths perceive CTPs as harmful to health, they simultaneously perceive ENDS, such as e-cigarettes, as less harmful [2,19,85,86,87]. Thus, these mixed results suggest that future research should unpack the differential perceptions of the use of CTPs and ENDS and that more education is needed about the harm of both CTP and ENDS use among adolescents. 

Social determinants have been identified as important predictors of health behaviors and outcomes [64,65]. In this regard, one study found that in an Appalachian population of middle and high school students, those who received tobacco information from families and friends were more likely to be e-cigarette and poly-tobacco users [57]. However, while extensive research has been conducted on social determinants for the use of CTPs [62,88], very little is known about ENDS use [57]. Due to the constraints/limitations imposed by school administrators and parents, this study was only able to collect data on two potential social determinants of ENDS use: belief that e-cigarettes are easy to obtain and parental/guardian advice. Approximately 80.4% of ever e-cigarette users and 88.7% current e-cigarette users reported that access to e-cigarettes was easy, although the association with e-cigarette use was not statistically significant. This high reported ease of access to e-cigarettes suggests the importance of the development and implementation of policies such as Tobacco 21 that prohibits adolescents from access to tobacco products including ENDS. For the issues of parental/guardian advising against the use of tobacco products, while 55.1% of ever e-cigarette users and 49.1% of current e-cigarette users reported that they had received such advice. Receiving parental/guardian advice about the dangers of tobacco products significantly reduced the likelihood of ever using an e-cigarette but was not statistically significant with being a current e-cigarette user. Because growing evidence indicates that ENDS use leads to the use of CTPs [7,9], this result of advice from parents/guardians against use of tobacco products can be generalized to ENDS use initiation among adolescents as a protective factor. Therefore, in an environment of high tobacco use prevalence, history of tobacco production, and complex sociocultural dynamics (as symbolized by the role of parents in the conduct of this study) parental/guardian advice could be critical to the health behaviors of adolescents. The report that only about half of ever and current e-cigarette users received parental/guardian advise against tobacco use indicates that the public health community in the region may not be involving parents/guardians in efforts to address the problem of tobacco use and highlights the need for outreach to parents/guardians in any initiative to prevent ENDS use among adolescents.

### Limitations of the Study

This study involved school-going adolescents in a highly homogenous population in an economically distressed, rural county in Appalachian Tennessee. While the findings could be extrapolated to the entire Appalachian Tennessee region and to some extent the larger Central Appalachian region, it is problematic to generalize nationwide. Further, this cross-sectional exploratory study could not establish causation due to the relatively small sample size. Additionally, the study asks adolescents to self-report of an “illegal” activity without any means of validation. As such, the study is subject to social desirability bias that may have contributed to underestimation of ever and current e-cigarette use. Lastly, this study was highly constrained by the demographic, familial, psychosocial, and SDOH information collected with the questionnaire, although prior studies have identified psychosocial and socioeconomic variables that predisposes adolescents to ENDS use [2,19]. This research was undertaken in an environment where participation in research is historically low [89]. Barriers to research within the rural county included sociocultural impediments (e.g., a high distrust for outsiders) and severe scrutiny of research by school administrators and parents [60,90]. As such, we were precluded from asking important questions pertaining to familial relations and social determinants of health behaviors, which are key factors associated with ENDS use. Despite these limitations, this study provides rare insights into ENDS usage and behaviors of adolescents in a distressed, rural county in Appalachian Tennessee that is an fundamental part of the “Tobacco Nation” [53]. As such, this study provides a foundation for a much larger study of the region and informs efforts to address disparities in tobacco use, a goal of Healthy People 2020 [91].

## 5. Conclusions

The use of ENDS has proliferated across all demographic and socioeconomic groups and geographic areas, and among youth, it has surpassed CTP use. This study adds to the stark paucity of studies involving adolescents in economically distressed, rural areas of the country. This study provides rare insights into adolescent ENDS use and has the potential to inform initiatives to address ENDS use among school-going adolescents in high-risk, economically distressed, rural environments. The study provides the foundation for much larger studies, which include queries about individual, family, psychosocial, and SDOH in order to understand ENDS among adolescents in the Appalachian region, aptly named Tobacco Nation.

## Figures and Tables

**Table 1:** Characteristics of study participants (*N* = 399).

Variable	Total *N* (%)	Ever Use of E-Cigarettes (%)	Chi-Square *p*-Value	Current Use of E-Cigarettes (%)	Chi-Square *p*-Value
Susceptible to e-cigarette use			<0.0001		<0.0001
No	249 (70.9)	27.1		7.6	
Yes	102 (29.1)	72.9		94.5	
Age of tobacco use initiation	109 (27.3)	34.5	**	35.1	**
Current age	339	30.6	**	15.5	**
Sex			0.3018		0.1942
Female	185 (52.7)	57.0		60.4	
Male	166 (47.3)	43.0		39.6	
School grade			0.0023		0.007
8th grade	177 (50.4)	38.3		30.2	
9th grade	174 (49.6)	61.7		69.8	
Perceive tobacco products are dangerous			0.0147		0.9943
Disagree	85 (24.2)	32.7		24.5	
Agree	266 (75.8)	67.3		75.5	
Believe e-cigarettes are easy to obtain			<0.0001		<0.0001
Not easy at all	133 (38.8)	19.6		11.3	
Easy to obtain	210 (61.2)	80.4		88.7	
Parental/Guardian advise against tobacco use			0.2875		0.7911
No	172 (49.0)	44.9		50.9	
Yes	179 (51.0)	55.1		49.1	

** The variables are continuous and therefore cannot be analyzed with the Chi-square test.

**Table 2:** Factors associated with ever use and current use of e-cigarettes among school-going youths in rural Appalachia (*N* = 399).

	Adjusted Values (Ever Use of E-Cigarette)			Adjusted Values (Current Use of E-Cigarette)		
Variable	OR	95% CI	*p*-Value	OR	95% CI	*p*-Value
Susceptible to e-cigarette use		0.03–0.25	<0.001		**0.01–0.12**	**<0.0001**
No	1			**1**		
Yes	0.09			**0.03**		
Age of tobacco use initiation	0.69	0.67–1.32	<0.0001	**0.89**	**0.83–0.97**	**0.0051**
Current age	0.94	0.67–1.32	0.7253	0.85	0.63–1.14	0.2720
Sex		0.33–2.39	0.8113		0.55–3.27	0.5122
Female	1			1		
Male	0.89			1.35		
Perceive tobacco products are dangerous	1	0.79–7.71	0.1182	**1**	**0.16–1.24**	**0.1203**
Disagree Agree	2.47			**0.45**		
Believe e-cigarettes are easy to obtain		0.25–2.18	0.5753		0.11–1.21	0.0999
Not easy at all	1			1		
Easy to obtain	0.73			0.36		
Parental/Guardian advise against tobacco use		0.16–1.34	0.1525		0.51–3.36	0.5723
No	1			1		
Yes	0.46			1.31		

Bold numbers are significant in the multivariable regression. OR, odds ratio; CI, confidence interval.
